# Whole‐Brain Monosynaptic Inputs and Outputs of Corticotropin‐Releasing Hormone Neurons of the Paraventricular Nucleus of the Hypothalamus in Male Mice

**DOI:** 10.1002/cns.71127

**Published:** 2026-08-30

**Authors:** Ziteng Yue, Danyang Zhao, Xinxin Chen, Yousheng Zhang, Heng Zhang, Yinchang Wang, Yuhang Liu, Yuxin Yang, Can Geng, Yimeng Song, Xiaoyi Wang, Sheng Wang

**Affiliations:** ^1^ Department of Neurobiology Hebei Medical University Shijiazhuang China; ^2^ Hebei Key Laboratory of Brain Science and Brain‐Inspired Intelligence Shijiazhuang China; ^3^ Beijing Key Laboratory for Sleep Breathing Disorder Beijing China

**Keywords:** corticotropin‐releasing hormone, inputs, outputs, paraventricular nucleus of the hypothalamus, stress

## Abstract

**Objective:**

The paraventricular nucleus of the hypothalamus (PVN) is a central hub integrating neuroendocrine, autonomic, and behavioral responses. Corticotropin‐releasing hormone (CRH) neurons in the PVN (PVN^CRH^) play a pivotal role in stress responses, anxiety, and motivation. However, the brain‐wide monosynaptic input–output architecture of PVN^CRH^ neurons remains incompletely defined, limiting circuit‐based functional dissection.

**Methods:**

Using Cre‐dependent modified rabies virus for retrograde monosynaptic tracing and adeno‐associated viruses for anterograde tracing in CRH‐Cre mice, we systematically mapped the whole‐brain inputs and outputs of PVN^CRH^ neurons.

**Results:**

PVN^CRH^ neurons received direct inputs from 55 brain regions, with a predominant contribution from diencephalon areas and an overall ipsilateral bias (61.4%). Notably, highly specific inputs originated from the ventral hippocampal CA1 and nucleus accumbens core. PVN^CRH^ neurons also sent widespread axonal projections throughout the brain. These outputs converge onto multiple circuit modules, with dense projections to emotion‐related nuclei and autonomic regulatory centers.

**Conclusion:**

This study establishes a comprehensive whole‐brain monosynaptic input–output atlas of PVN^CRH^ neurons. The resultant neuroanatomical framework delineates their extensive connectivity with stress‐, emotion‐, autonomic‐, and respiration‐related circuits, providing a structural basis for future circuit‐level investigations into stress‐related psychiatric disorders and systemic autonomic dysregulation.

## Graphical abstract

Using rabies‐based monosynaptic retrograde tracing and AAV anterograde labeling, this study maps the brain‐wide inputs and outputs of PVN CRH neurons, identifying them as a key hub coordinating stress, autonomic, and behavioral responses.

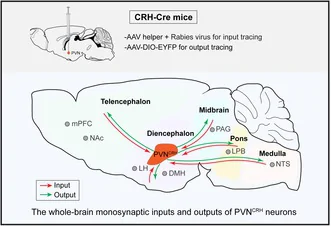

## 1 Introduction

The paraventricular nucleus of the hypothalamus (PVN) is a key integrative center that coordinates neuroendocrine, autonomic, and behavioral responses, thereby maintaining homeostasis and mediating adaptation to stress [1, 2]. The PVN contains functionally heterogeneous neuronal subpopulations, including corticotropin‐releasing hormone (CRH), oxytocin (OXT), arginine vasopressin (AVP), and melanocortin 4 receptor (MC4R) neurons [3, 4, 5, 6, 7]. Through distinct projection pathways, these subtypes regulate the hypothalamic–pituitary–adrenal (HPA) axis, sympathetic and parasympathetic outflow, feeding and energy metabolism, thermoregulation, cardiovascular function, and emotional behaviors [8, 9, 10, 11, 12]. Understanding how specific subpopulations of PVN neurons are embedded within whole‐brain circuits is therefore essential for elucidating their roles in both physiological and pathological conditions.

Recent advances in cell type‐specific viral tracing have enabled systematic mapping of inputs and outputs for defined PVN neuronal populations. Monosynaptic rabies and Cre‐dependent AAV tracings have shown that OXT‐expressing PVN (PVN^OXT^) neurons primarily receive inputs from local hypothalamic nuclei such as the lateral hypothalamus (LH), zona incerta (ZI), and the thalamic regions, while sending widespread projections to the midbrain, pons, and telencephalon to modulate social behavior, pain, and anxiety [13, 14, 15, 16]. AVP‐expressing PVN (PVN^AVP^) neurons receive concentrated inputs from osmo‐ and thermo‐regulatory structures, including the vascular organ of the lamina terminalis (OVLT), medial preoptic nucleus (MnPO), and the dorsomedial and ventromedial hypothalamus (DMH, VMH). Their output patterns are sexually dimorphic and contribute to blood pressure regulation [17, 18]. MC4R‐expressing PVN (PVN^MC4R^) neurons participate in feeding and cardiovascular control via projections to the parabrachial nucleus (PBN), nucleus tractus solitarius (NTS), and rostral ventrolateral medulla (RVLM) [6]. These findings highlight that distinct subsets of PVN neurons are embedded in partially segregated yet overlapping circuits that support specific physiological functions.

Among these subpopulations, CRH‐expressing PVN neurons (PVN^CRH^) are abundant and represent the principal hypothalamic subpopulation that initiates the HPA axis. In addition to their classical endocrine role, recent work indicates that PVN^CRH^ neurons directly modulate anxiety, reward, autonomic functions, and stress‐related behaviors via co‐transmission of fast neurotransmitter such as glutamate [19, 20, 21]. For example, PVN^CRH^ neurons projecting to the ventral tegmental area (VTA) influence reward‐related behavior [22], while projections to the NTS and RVLM contribute to cardiorespiratory reflexes and blood pressure regulation [23, 24]. Furthermore, their projections to the dorsal motor nucleus of the vagus (DMNV) regulate gastric motility [25]. Moreover, PVN^CRH^ neuronal activity can exert protective effects in acute lung injury through a sympathetic–neutrophil axis [26]. PVN^CRH^ neuronal dysfunction has been implicated in depression, hypertension, pain‐related anxiety, and substance use disorders [23, 27, 28, 29]. Despite extensive functional studies, a comprehensive and quantitative whole‐brain map of monosynaptic inputs and outpus of PVN^CRH^ neurons is still lacking.

In the present study, we combined Cre‐dependent monosynaptic rabies tracing with anterograde AAV‐based tracing in CRH‐Cre mice to systematically map the whole‐brain connectome of PVN^CRH^ neurons. We identified 55 brain regions providing direct inputs and delineated the major downstream projection targets across telencephalic, diencephalic, midbrain, pontine, and medullary structures. By defining the input–output architecture of PVN^CRH^ neurons at cellular resolution, this work provides an anatomical framework for understanding how these neurons integrate stress‐ and state‐related signals and for guiding future circuit‐level investigations into stress‐related psychiatric and cardiometabolic disorders.

## 2 Materials and Methods

### 2.1 Animals

Adult male CRH*‐IRES‐Cre* mice (stock No. 012704, Jackson Laboratory, 8–10 weeks old, 23–28 g) and adult male C57 BL/6J mice (Beijing Vital River Laboratory Animal Technology, Beijing, China, 8–10 weeks old, 23–28 g) were used for viral tracing. CRH‐Cre mice can specifically express Cre recombinase under the control of its gene promoter. Mice were randomly allocated into two experimental cohorts: the retrograde monosynaptic tracing group (*n* = 5) and the anterograde tracing group (*n* = 3). Animals were group‐housed under controlled conditions (23°C ± 1°C, 50% ± 10% relative humidity) with a 12:12 h light/dark cycle (lights on at 7:00 a.m.) and ad libitum access to food and water. Every effort was made to minimize animal usage and suffering. Mice with misplaced injection virus injections or abnormal viral expression were excluded from subsequent data analysis.

### 2.2 Viruses and Surgeries

All viruses were packaged and purchased from Brain Case (Wuhan, China) and stored at −80°C before use. All procedures involving the viruses were conducted under strict sterile conditions to avoid contamination and repeated freeze–thaw cycles. The titers of the Cre‐dependent AAV vectors (AAV‐EF1α‐DIO‐TVA‐GFP, AAV‐EF1α‐DIO‐RG, and rAAV9‐EF1α‐DIO‐EYFP‐WPRE) were approximately 2 × 10^12^ genome copies/mL, and the modified rabies virus (RV‐EnvA‐ΔG‐tdTomato) was 2 × 10^8^ genome copies/mL.

Mice were anesthetized with pentobarbital sodium (60 mg/kg, i.p.), supplemented as needed (30% of the initial dose), and fixed onto a stereotaxic apparatus (RWD Life Science, China) for all stereotaxic viral injections.

For retrograde tracing experiments, a 2:1 mixture of AAV‐EF1α‐DIO‐TVA‐GFP and AAV‐EF1α‐DIO‐RG (60 nL) was injected unilaterally into the PVN (coordinates: anterior–posterior (AP) = −0.5 mm, medial‐lateral (ML) = ±0.17 mm, dorsal‐ventral (DV) = −4.9 mm) at a rate of 20 nL/min. After injection, the pipette was left in place for 10 min to allow sufficient virus diffusion, and then slowly withdrawn. Two weeks post‐injection, RV‐EnvA‐ΔG‐tdTomato (60 nL) was injected into the same PVN coordinate. Mice were allowed to survive for an additional 1 week before perfusion.

For anterograde tracing experiments, rAAV‐EF1α‐DIO‐EYFP‐WPRE (60 nL) was injected unilaterally into the PVN using the same coordinates and procedures described above. Mice were allowed to recover for 4 weeks to ensure adequate viral expression and axonal transport before perfusion.

Postoperative care included the administration of ibuprofen (5 mg/kg, i.p.). Mice were warmed until full recovery from anesthesia, and their body weight, general behavior, food and water intake were closely monitored daily for 1 week after surgery.

### 2.3 Histology and Immunofluorescence

At the end of the viral tracing survival period, mice were deeply anesthetized with pentobarbital sodium (60 mg/kg, i.p.) and transcardially perfused with saline until the liver and cardiac atria turned pale white, followed by ice‐cold 4% paraformaldehyde (PFA) in 0.1 M phosphate buffer (PB, pH 7.4). Brains were carefully extracted, post‐fixed in 4% PFA at 4°C for 24 h, and cryopreserved sequentially in 10%, 20%, and 30% sucrose in 0.1 M PB (pH 7.4) at 4°C until they sank. Tissues were embedded in optimal cutting temperature (OCT) compound and stored at −80°C until sectioning. Coronal brain sections (25 μm thickness) were cut on a cryostat (Leica CM1950) from the olfactory bulb to the medulla oblongata and collected in four series in 0.01 M phosphate‐buffered saline (PBS, pH 7.4).

For immunofluorescence staining, free‐floating sections were washed 6 times in 0.01 mol/L PBS and incubated overnight at 4°C with primary antibodies diluted in PBS containing 0.25% Triton X‐100 (T9284; Sigma‐Aldrich, USA) and 5% Bovine Serum Albumin (9048‐46‐8; Sigma‐Aldrich, USA). The primary antibody used was chicken anti‐GFP (1:1000, catalog #ab13970, RRID:AB_300798, Abcam). The secondary antibody used was goat anti‐chicken IgY H&L (Alexa Fluor 488) (1:1000, catalog #ab150169, RRID:AB_2636803, Abcam). Sections were then rinsed, mounted on glass slides, and cover‐slipped with DAPI Fluoromount‐GTM (0100‐20; Southern Biotech, USA).

### 2.4 RNAscope Fluorescence In Situ Hybridization

To identify the neuronal type within the PVN, brain sections were collected 4 weeks after viral injection and processed using RNAscope‐Fluorescence in situ Hybridization (RNAscope‐FISH), following previously described protocols [30, 31]. The mice were deeply anesthetized with urethane (1.8 g/kg, intraperitoneally). After the loss of the toe‐pinch reflex, transcardial perfusion with ice‐cold saline was performed. The animals were then fixed with 4% paraformaldehyde (PFA) at 4°C, and the brains were harvested and post‐fixed in the same PFA solution for 24 h at 4°C. Subsequently, the tissue was dehydrated by immersion in 15% and then 30% sucrose in phosphate‐buffered saline (PBS), embedded in OCT compound, and stored at −80°C. Coronal sections of 25 μm thickness were cut using a cryostat (CM1950, Leica Microsystems, Wetzlar, Germany). The RNAscope multiplex fluorescent assay was performed on the slides according to the manufacturer's manual (document no. 323100‐USM). All RNAscope‐FISH probes were designed and validated by Advanced Cell Diagnostics Inc. Images were acquired with a confocal microscope (LSM 800; Carl Zeiss, Jena, Germany) and processed using ZEN software. Cells were identified and classified with the ImageJ cell counter plugin. Neurons expressing *Crh* mRNA (probe Cat#316091, Advanced Cell Diagnostics, USA) were visualized in channel C1, and those expressing *GFP* mRNA (probe Cat#400281, Advanced Cell Diagnostics, USA) in channel C3. Expression was scored as binary (yes/no) based on predefined criteria: the presence of at least five distinct punctate fluorescent signals associated with a DAPI‐stained nucleus (DAPI Fluoromount‐G, Cat#SBA‐0100‐20, SouthernBio, China). Cells that met this criterion in both channels were considered double‐labeled, indicating colocalization.

### 2.5 Imaging

Whole brain slide scanning was performed using a virtual microscopy slide scanning system (Zeiss LSM 800, Carl Zeiss, Germany) equipped with an EC Plan‐NEOFLUAR 10×/0.3 NA M27 objective. Images were acquired at 10× magnification to visualize the distribution of tdTomato‐labeled presynaptic input neurons (retrograde tracing) and EYFP‐labeled axonal projections (anterograde tracing). For standardized grayscale display and quantitative accuracy, all whole‐brain slice scanning images were converted to 8‐bit grayscale and adjusted with a consistent threshold using ImageJ software before subsequent image processing.

High‐resolution confocal images of starter cells were acquired using a confocal laser scanning microscope (Zeiss LSM 800, Carl Zeiss, Germany) with a 20× objective. Brain region localization of labeled neurons was performed by matching sections to *The Mouse Brain in Stereotaxic Coordinates*, 4th edition (Paxinos and Franklin), with reference to DAPI nuclear staining and anatomical landmarks. Images were processed and assembled using Adobe Illustrator 2025 (Adobe Inc., San Jose, CA, USA). Semi‐automated quantification of labeled neuron numbers and cell body density was performed using ImageJ software.

### 2.6 Data Analysis and Statistics

First, to define the exact areas for quantification, we adopted the method described by Liu et al. [32]. Specifically, coronal images were aligned to *The Mouse Brain in Stereotaxic Coordinates* [33] and the boundaries of each brain region were manually delineated to generate Regions of Interest (ROIs). Quantification within each ROI was then performed using the software Fiji and a semi‐automated pipeline. The “semi‐automated” workflow is defined as follows:

Automated Step (Parameters): The images were converted to 8‐bit, and a thresholding algorithm was applied to distinguish fluorescent signals from the background. We then used the software's built‐in counting function (e.g., “Analyze Particles”). The parameters were strictly set to a pixel area of 50–500 μm^2^ and a circularity of 0.4–1.0 to isolate somata and exclude non‐specific puncta or artifacts. Puncta‐like fluorescent signals were quantified by measuring their relative size, and the number and occupied area were calculated using Fiji.

For retrograde monosynaptic tracing experiments, tdTomato‐positive presynaptic input neurons were quantified in every fourth coronal section throughout the entire rostrocaudal extent of the brain to avoid duplicate counting. For each animal, the number of tdTomato‐positive neurons assigned to each anatomically defined brain region was divided by the total number of tdTomato‐positive input neurons counted across the whole brain and expressed as a percentage. Thus, the relative input proportion for each region was calculated as: (number of tdTomato‐positive neurons in a given brain region/total number of tdTomato‐positive input neurons counted across the whole brain) × 100%.

This value represents the contribution of each brain region to the total labeled input population and does not account for the total number of neurons or cells within individual coronal sections.

This measure represents the proportional contribution of each region to the total labeled input population and was not normalized to the total number of neurons or cells within each coronal section.

For anterograde tracing experiments: The relative projection proportion of each brain region was calculated as follows: (fluorescent area of EYFP‐positive axons in the target brain region/total fluorescent area of EYFP‐positive axons across the whole brain) × 100%. The projection density was graded into four levels according to the projection proportion: numerous (> 4%), substantial (2%–4%), moderate (1%–2%), and sparse (< 1%).

For the statistical analysis, the data were imported into Prism 9.0.0 (GraphPad) for analysis. All data were presented in the form of mean ± standard error. No data outliers were excluded in this study. Some visualizations were conducted using the R program (version 4.5.2).

## 3 Results

### 3.1 Experimental Strategy for Targeted Labeling of PVN^CRH^ Neurons

**FIGURE 1:**
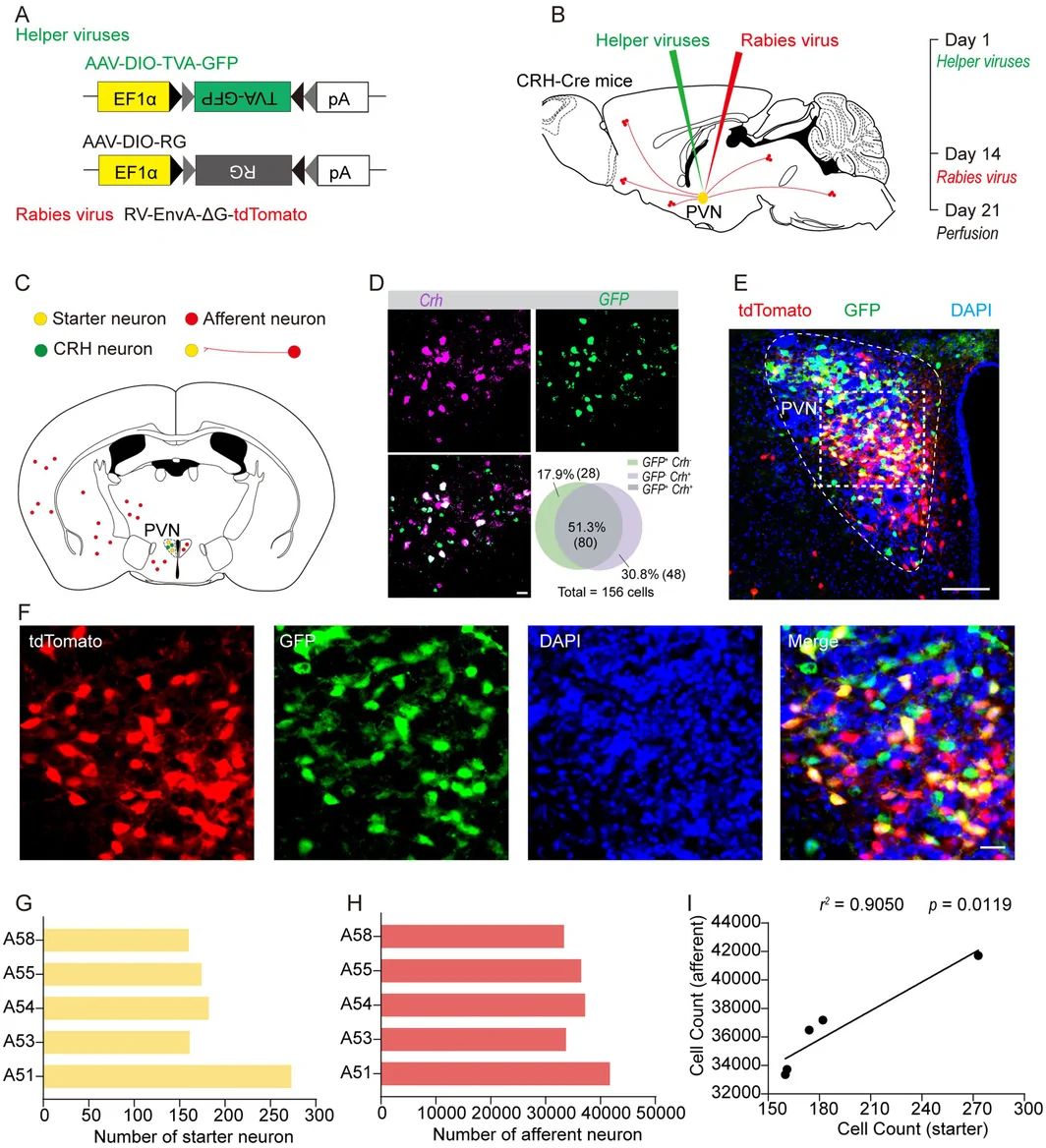
Trans‐synaptic rabies retrograde tracing of monosynaptic inputs to PVN^CRH^ neurons. (A) Schematic of the viral vectors for RV‐based trans‐synaptic retrograde tracing, including helper viruses with Cre‐dependent expression of TVA receptor (AAV‐EF1α‐DIO‐TVA‐GFP) and RG (AAV‐EF1α‐DIO‐RG). The RV was genetically modified by pseudotyping with EnvA (RV‐EnvA‐∆G‐tdTomato). (B) Timeline of the viral injection strategy and experimental procedure in CRH‐Cre mice. (C) Diagram illustrating the logic of monosynaptic retrograde tracing. CRH neurons (green) expressing TVA and RG are infected by RV to become starter neurons (yellow), which then label their direct upstream afferent neurons (red). (D) RNAscope‐FISH images demonstrating the colocalization of *GFP* (green) and *Crh* (pink) mRNAs (*n* = 156 cells). Scale bar, 20 μm. (E) Immunohistochemical imaging showed the expression of RV‐labeled neurons (red) in the PVN from CRH‐Cre mice. Scale bar, 100 μm. (F) Photomicrograph showed that the starter neurons (yellow) were infected with AAV helper virus and RV. Scale bar, 20 μm. (G) The total number of starter neurons in the PVN for each of the five mice (animal IDs: A51, A53, A54, A55, and A58) with confirmed accurate viral targeting. (H) The total number of afferent neurons for each of the five mice (animal IDs: A51, A53, A54, A55, and A58) with confirmed accurate viral targeting. (I) There was a linear relationship between starter and input neurons across all samples, indicating that the virus had the same trans‐synaptic efficiency in different samples (*n* = 5 mice).

To establish the whole‐brain input atlas of PVN^CRH^ neurons, we employed a glycoprotein (G)‐deleted rabies virus (RV‐ΔG) retrograde tracing strategy combined with the Cre‐LoxP system (Figure 1A). Initially, a Cre‐dependent helper virus cocktail (AAV‐DIO‐TVA‐GFP and AAV‐DIO‐RG) was unilaterally injected into the PVN of CRH‐Cre mice. Two weeks post‐injection, RV‐EnvA‐ΔG‐tdTomato was administered at the same coordinates (Figure 1B). This virus specifically infects TVA‐expressing PVN^CRH^ neurons via the EnvA pseudotype and spreads retrogradely across single synapses to upstream presynaptic neurons only in the presence of the G‐protein provided by the helper virus (Figure 1C). We injected helper viruses and rabies virus into the PVN in 8 mice and observed that the viral transfection was confined to the PVN in 5 of 8 mice. This ruled out the interference caused by inaccurate viral transfection sites. RNAScope‐FISH analyses confirmed that the *GFP* ^+^ and *Crh* ^+^ co‐labeled neurons accounted for 51.3% of the total counted neurons. (Figure 1D). Moreover, immunofluorescence analysis revealed that starter cells (co‐expressing GFP and tdTomato, composite color) were strictly confined within the boundaries of the PVN (Figure 1E). The majority of tdTomato‐labeled presynaptic neurons was also identified within the PVN itself (Figure 1F), indicating the presence of an intra‐PVN microcircuit, wherein local interneurons or adjacent non‐CRH neurons provide monosynaptic inputs to PVN^CRH^ neurons. In contrast, using the same viral strategy in wild‐type mice, we detected no neurons expressing GFP or tdTomato in the PVN, confirming the Cre‐dependency and specificity of the tracing system (Figure S1A,B). To assess inter‐animal variability (*n* = 5 mice) using the tracing approach, we quantified the number of starter neurons and total tdTomato‐labeled input neurons in each mouse (Figure 1G,H). We then performed a regression analysis between starter and input neuron counts (Figure 1I), which revealed a positive linear relationship across animals. This scaling indicates that, under current conditions, the virus set produced a consistent dependence of total retrograde labeling on the number of starter cells, supporting the stability and reproducibility of the retrograde tracing across samples. All subsequent input analyses were therefore expressed as proportions of total input neurons per animal to minimize the influence of absolute starter cell number on regional input profiles.

### 3.2 Brain‐Wide Input Architecture of PVN^CRH^ Neurons

**FIGURE 2:**
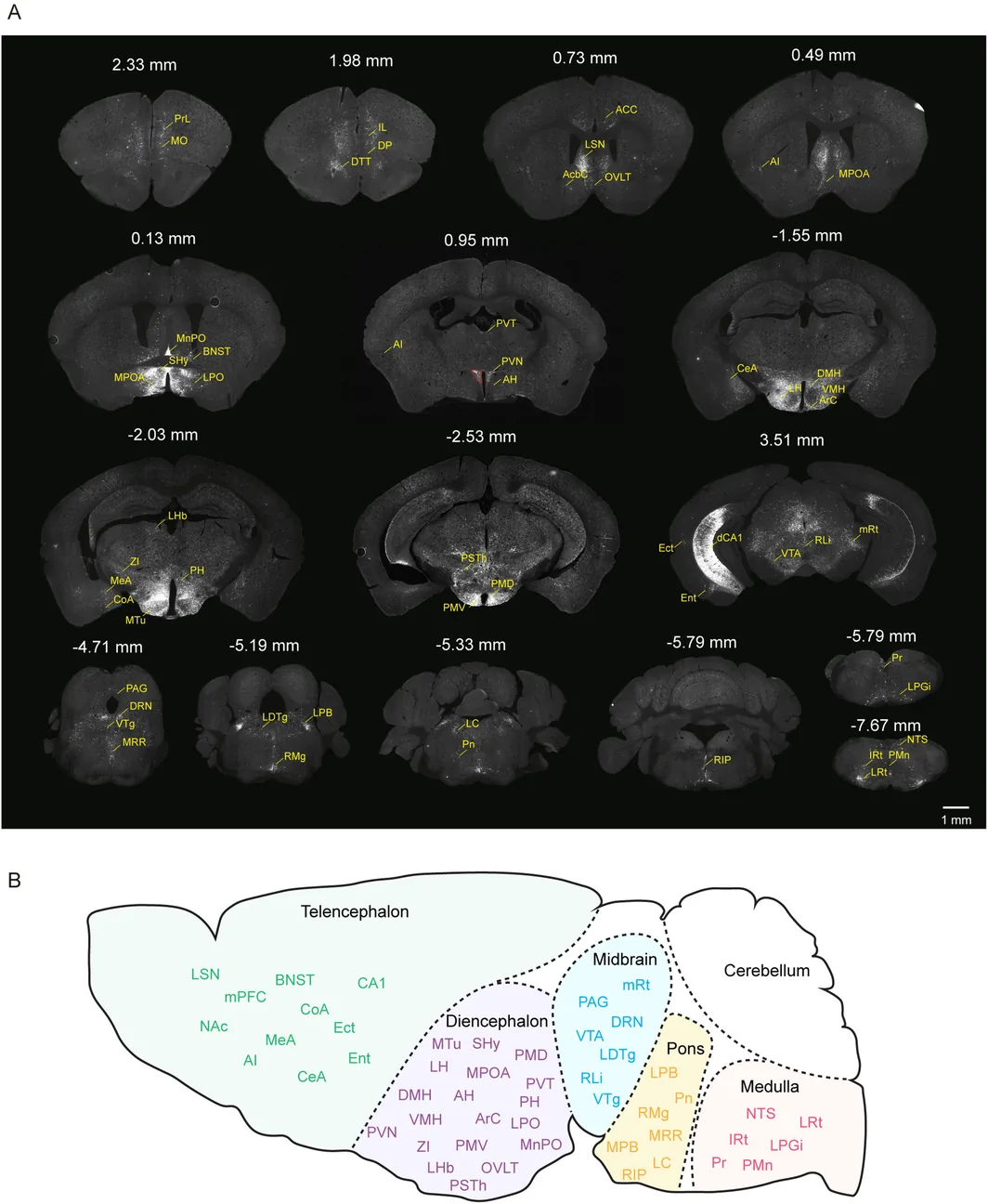
Whole‐brain distribution of monosynaptic inputs to PVN^CRH^ neurons. (A) Whole‐brain monosynaptic inputs to PVN^CRH^ neurons were visualized via retrograde viral tracing. Brain regions were identified and annotated according to the standard mouse brain atlas: *The Mouse Brain in Stereotaxic Coordinates*, 4th edition (Paxinos and Franklin). Representative coronal sections show the distribution of upstream input neurons (white) across the entire brain, spanning from the anterior forebrain to the posterior brainstem. Numbers indicate the anterior–posterior (AP) coordinate (in mm) relative to the Bregma. Scale bar: 1 mm. (B) Schematic summary of monosynaptic input connectivity of PVN^CRH^ neurons. AcbC, accumbens nucleus, core; ACC, accessory neurosecretory nuclei; AH, anterior hypothalamic area; AI, agranular insular cortex; ArC, arcuate hypothalamic nucleus; BNST, bed nucleus of the stria terminalis; CeA, central amygdaloid nucleus; CoA, cortical amygdaloid nucleus; DMH, dorsomedial hypothalamic nucleus; DP, dorsal peduncular cortex; DRN, dorsal raphe nucleus; DTT, dorsal tenia tecta; Ect, ectorhinal cortex; Ent, entorhinal cortex; IL, infralimbic cortex; IRt, intermediate reticular nucleus; LC, locus coeruleus; LDTg, laterodorsal tegmental nucleus; LH, lateral hypothalamic area; LHb, lateral habenula; LPB, lateral parabrachial nucleus; LPGi, lateral paragigantocellular nucleus; LPO, lateral preoptic area; LRt, lateral reticular nucleus; LSN, lateral septal nucleus; MeA, medial amygdaloid nucleus; MnPO, median preoptic nucleus; MO, medial orbital cortex; MPB, medial parabrachial nucleus; MPOA, medial preoptic area; MRR, median raphe nucleus; mRt, medial raphe nucleus; MTu, medial tuberomammillary nucleus; NTS, nucleus of the solitary tract; OVLT, organum vasculosum of the lamina terminalis; PAG, periaqueductal gray; PH, posterior hypothalamic area; PMD, premammillary nucleus, dorsal part; PMn, paramedian raphe nucleus; PMV, premammillary nucleus, ventral part; Pn, pontine nuclei; Pr, perirhinal cortex; PrL, prelimbic cortex; PSTh, parasubthalamic nucleus; PVN, paraventricular nucleus of the hypothalamus; PVT, paraventricular thalamic nucleus; RIP, raphe interpositus nucleus; RLi, rostral linear nucleus of the raphe; RMg, raphe magnus nucleus; SHy, septohypothalamic nucleus; vCA1, ventral field CA1 of the hippocampus; VMH, ventromedial hypothalamic nucleus; VTA, ventral tegmental area; VTg, ventral tegmental nucleus; ZI, zona incerta.

To systematically map the whole‐brain synaptic inputs to PVN^CRH^ neurons, we analyzed consecutive coronal sections from five mice with starter cells confined to the PVN (Figure 2A, Figure S2). Based on anatomical organization, the brain was classified into five major divisions: telencephalon, diencephalon, midbrain, pons, and medulla oblongata. tdTomato‐labeled input neurons were widely distributed across these regions, with the exception of the cerebellum where no labeling was detected (Figure 2B).

**FIGURE 3:**
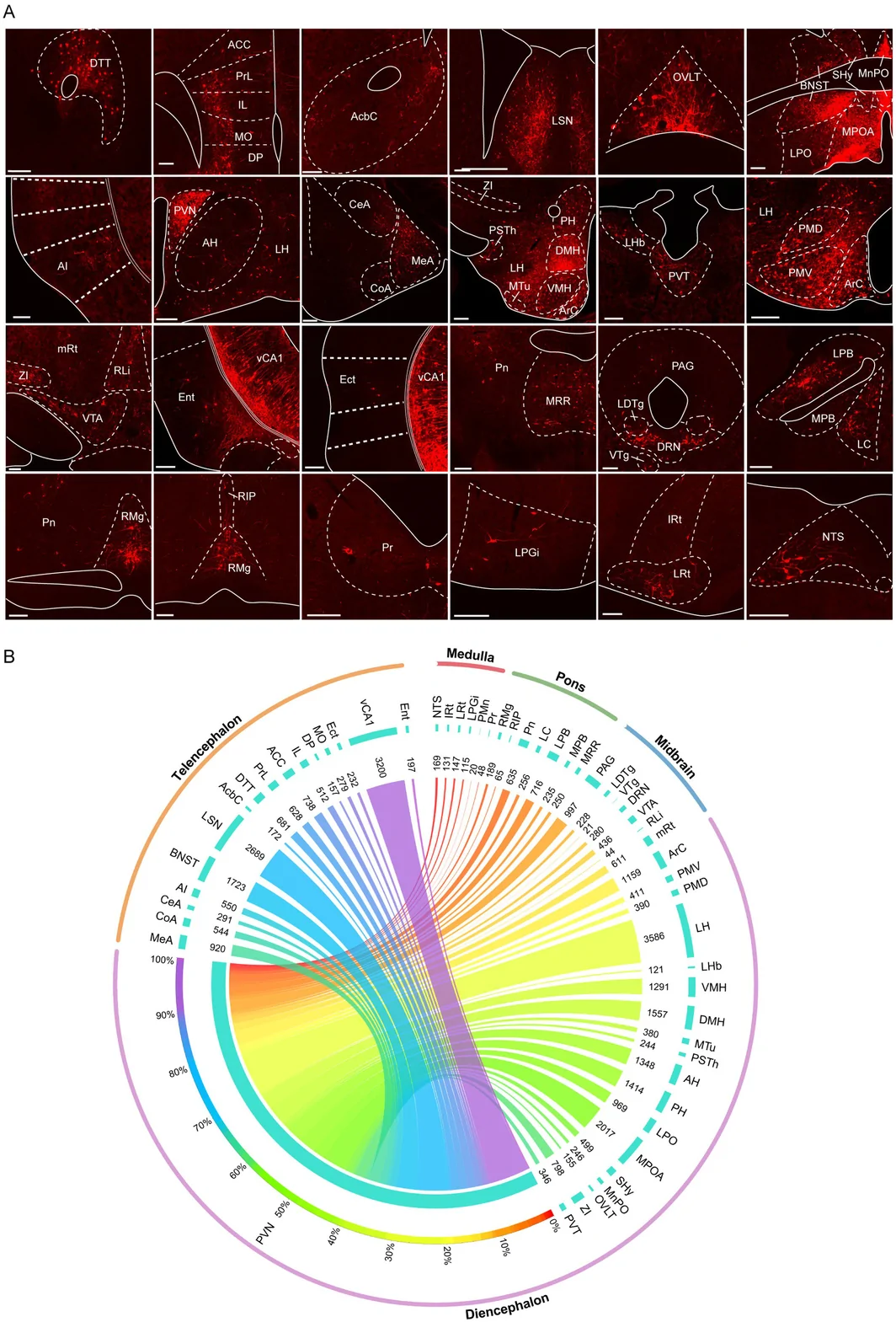
Distribution and quantitative analysis of monosynaptic inputs to PVN^CRH^ neurons. (A) Typical images of brain regions exhibiting monosynaptic inputs targeting PVN^CRH^ neurons. Fluorescent photographs showing tdTomato^+^ neurons (red) across various brain regions, indicating monosynaptic input to PVN^CRH^ neurons. Brain regions are outlined with dashed lines. Scale bars, 200 μm. (B) Circos plot of whole‐brain monosynaptic inputs to PVN^CRH^ neurons. Each outer sector represents an anatomically defined input region and is color‐coded by major brain division (telencephalon, diencephalon, midbrain, pons, and medulla). The second track indicates the relative proportion of total input neurons from each region and the radial length reflects the proportion of input. Links represent direct monosynaptic inputs from each upstream region to the PVN. Inner numeric labels indicate the absolute number of labeled presynaptic neurons in each region. Central ribbons connect the input regions to the starter region (PVN), with the ribbon thickness proportional to the relative input strength.

Representative images demonstrated that substantial input neurons were observed in several critical nuclei across coronal sections (Figure 3A). In the telencephalon, prominent inputs originated from the ventral field CA1 of the hippocampus (vCA1), lateral septal nucleus (LSN), and bed nucleus of the stria terminalis (BNST). Within the diencephalon, dense labeling was identified in circumventricular organs, such as OVLT and MnPO, and hypothalamic nuclei including PVN, DMH, VMH, LH, and arcuate hypothalamic nucleus (ArC), as well as thalamic regions containing lateral habenular nucleus (LHb) and paraventricular thalamic nucleus (PVT). In the midbrain, tdTomato‐positive neurons were concentrated in the VTA, periaqueductal gray (PAG), and dorsal raphe nucleus (DRN). The pontine regions showed labeling in the laterodorsal tegmental nucleus (LDTg), lateral parabrachial nucleus (LPB), and locus coeruleus (LC), while medullary inputs arose primarily from the NTS, intermediate reticular nucleus (IRt), and lateral paragigantocellular nucleus (LPGi).

To comprehensively analyze the input distribution, we generated a Circos plot that quantitatively mapped the synaptic inputs to PVN^CRH^ neurons across 55 anatomical regions (Figure 3B). The plot revealed a complex connectivity landscape with distinct input characteristics: the inner track displayed the absolute number of input neurons for each region, demonstrating a hierarchical input pattern. The proportion of afferent neurons was highest in the diencephalon, with the medial preoptic area (MPOA) and LH showing the most substantial neuronal contributions. Telencephalic regions, particularly the vCA1 and LSN, emerged as significant input sources. The midbrain, pontine, and medullary regions contributed progressively fewer input neurons. The central ribbons of the Circos plot illustrated the connectivity strength between input regions and the PVN^CRH^ neurons, with ribbon thickness directly proportional to the relative proportion of afferent neurons. This visualization highlighted the differential connectivity patterns across various brain regions, revealing the nuanced synaptic architecture of PVN^CRH^ neurons.

### 3.3 Quantitative Analysis of Lateralization of Whole‐Brain Inputs to PVN^CRH^ Neurons

**FIGURE 4:**
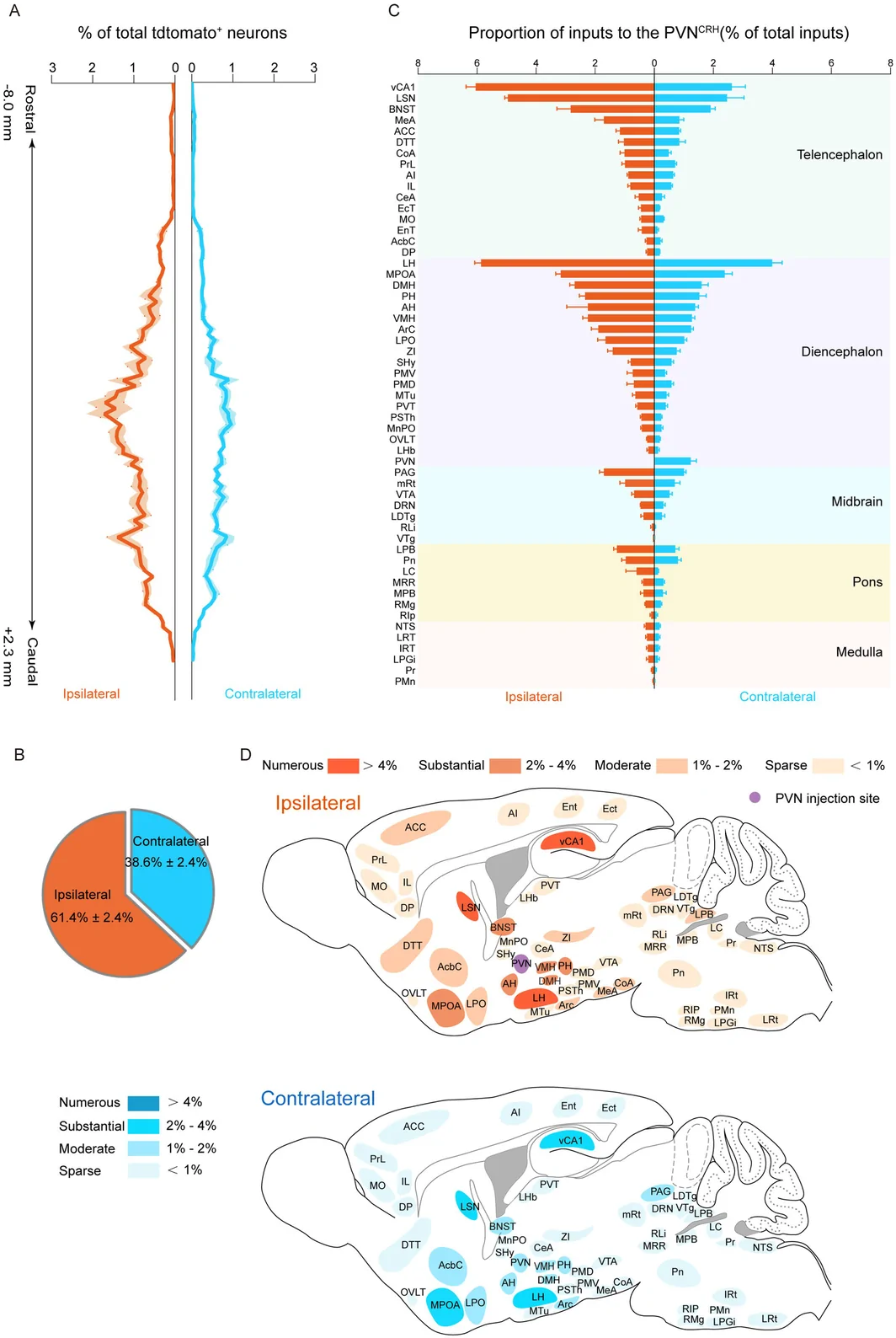
Quantitative analysis of ipsilateral and contralateral inputs to PVN^CRH^ neurons. (A) Descriptive rostro‐caudal distribution of labeled PVN‐projecting input neurons. For each coronal level, the number of labeled input neurons was expressed as a percentage of the total number of labeled input neurons detected across the brain. These values were not normalized to the total neuronal count within each coronal section and therefore should not be interpreted as relative input density. Inputs are plotted separately for ipsilateral (orange) and contralateral (blue) hemispheres. (B) Pie chart showing proportion of ipsilateral (61.4% ± 2.4%) and contralateral (38.6% ± 2.4%) inputs across the brain. (C) Proportions of presynaptic inputs to PVN^CRH^ neurons from various brain regions, expressed as a percentage of total inputs. Orange bars indicate ipsilateral inputs, and blue bars indicate contralateral inputs. Brain regions are categorized into major divisions: Telencephalon, diencephalon, midbrain, pons, and medulla oblongata. (D) Schematic of sagittal brain maps depicting distribution of ipsilateral (orange) and contralateral (blue) monosynaptic inputs to PVN^CRH^ neurons. Input strength from each region is categorized based on its proportion of the total inputs: Numerous (> 4%), Substantial (2%–4%), Moderate (1%–2%), or Sparse (< 1%). Purple dot represents PVN injection site. *n* = 5 mice. Data are presented as mean ± SEM.

To further describe the anatomical organization of monosynaptic inputs to PVN^CRH^ neurons, we analyzed the distribution of tdTomato‐labeled input neurons across 55 anatomically defined regions. We next examined how these labeled input neurons were distributed along the rostro‐caudal axis. For each animal, the number of labeled input neurons detected at each coronal level was expressed as a percentage of the total number of labeled input neurons in that animal. This analysis provides a descriptive measure of the rostro‐caudal distribution of labeled inputs, rather than a measure of preferential input density within each coronal plane. Using this measure, both hemispheres showed broadly similar distribution profiles, with a larger proportion of labeled input neurons located between approximately +1.0 and +2.3 mm relative to bregma. Within this range, the proportion of ipsilateral labeled inputs was generally higher than that of contralateral inputs (Figure 4A). Overall, 61.4% ± 2.4% of labeled input neurons were located ipsilateral to the injection site, whereas 38.6% ± 2.4% were found contralaterally, indicating a robust ipsilateral dominance while preserving substantial cross‐hemispheric innervation (Figure 4B).

Quantitative analyses revealed that input neurons were primarily distributed in the diencephalon, followed by the telencephalon, midbrain, pons, and medulla oblongata (Figure 4C). Within the telencephalon, the vCA1 appears to contribute the largest fraction of ipsilateral inputs relative to the injection side, with the LSN as the second major source. For contralateral inputs, the vCA1 and LSN provided approximately similar input proportions. In the diencephalon, ipsilateral and contralateral inputs were dominated by the LH, followed by the MPOA. Notably, the contralateral PVN also contained a considerable number of labeled neurons, indicating substantial reciprocal interactions between PVN^CRH^ neurons across hemispheres. In contrast, midbrain, pontine, and medullary inputs were relatively sparse, although within each division, the ipsilateral and contralateral distributions followed similar trends. To provide an intuitive overview of input strength, all afferent nuclei were further categorized into four tiers based on their contribution to the total input population: numerous (> 4%), substantial (2%–4%), moderate (1%–2%), and sparse (< 1%) (Figure 4D). Together, these lateralization analyses define a biased yet bilaterally organized input architecture onto PVN^CRH^ neurons, highlighting diencephalic and forebrain regions as dominant afferent sources.

### 3.4 Efferent Projection Profile of PVN^CRH^ Neurons

**FIGURE 5:**
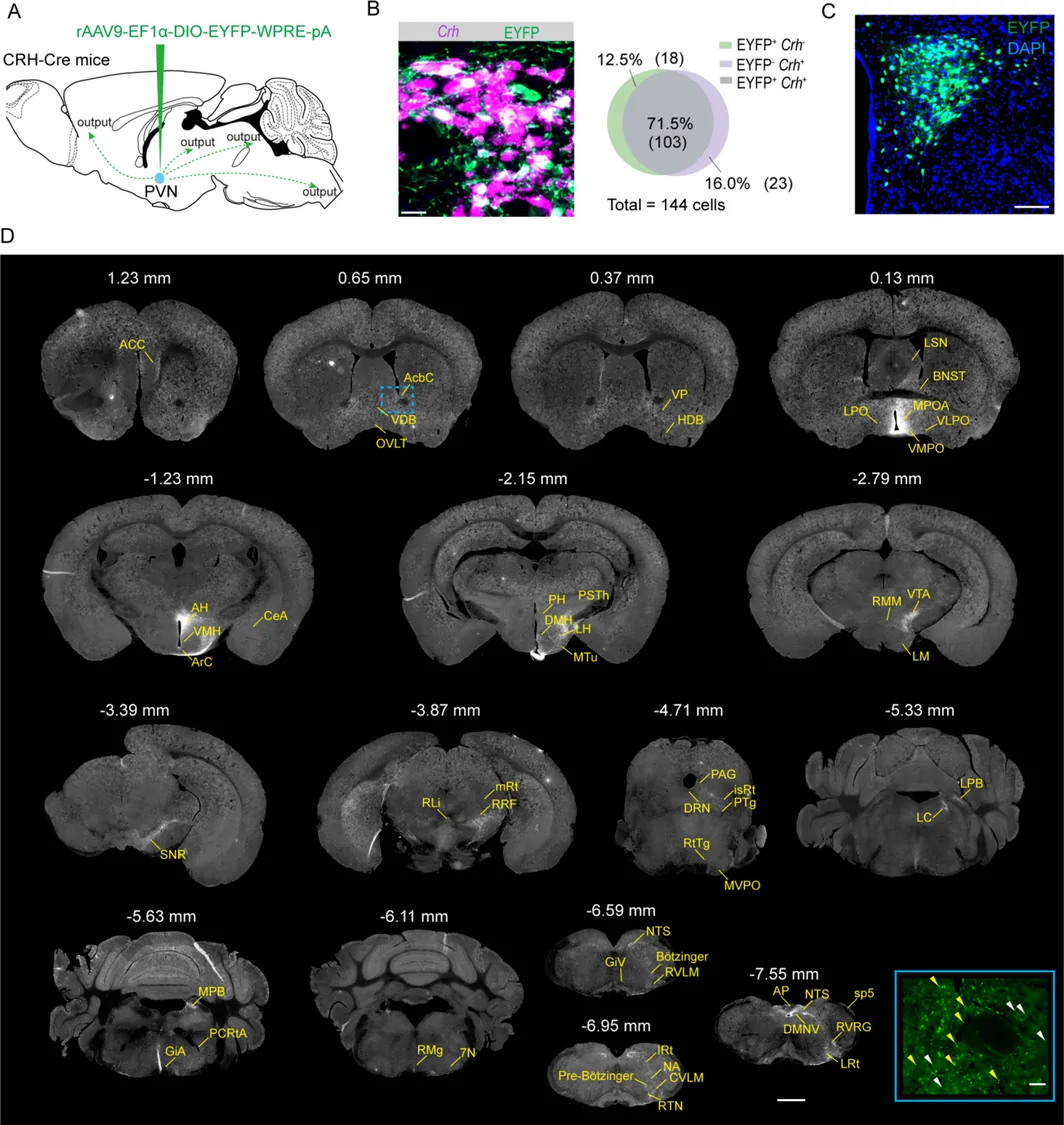
Whole‐brain mapping of efferent projection of PVN^CRH^ neurons. (A) Schematic illustration of a sagittal brain section depicting the whole‐brain efferent projection patterns (green) of PVN^CRH^ neurons and the unilateral injection site (blue circle) for rAAV‐EF1α‐DIO‐EYFP in CRH‐Cre mice. (B) RNAscope‐FISH images demonstrating colocalization of EYFP (green) and *Crh* (pink) mRNAs (*n* = 144 cells). Scale bar, 20 μm. (C) Representative immunohistochemical validation of the injection site in the PVN. Scale bar, 100 μm. (D) Representative coronal sections showing EYFP‐labeled axonal projections from PVN^CRH^ neurons across the entire rostrocaudal axis. Brain regions are labeled according to the standard mouse brain atlas *The Mouse Brain in Stereotaxic Coordinates*, 4th edition (Paxinos and Franklin). Numbers below each section indicate the anteroposterior (AP) coordinates (in mm) relative to Bregma. Scale bars: 1 mm for the main panels. The inset at the bottom right corner (blue solid rectangle) is a magnified view of the region indicated by the blue dashed rectangle in the 0.65 mm section (scale bar: 100 μm). In the inset, yellow arrowheads point to fluorescent puncta‐like enlargements along labeled fibers, whereas white arrowheads indicate fibers of passage. 7N, facial nucleus; AcbC, accumbens nucleus, core; ACC, accessory neurosecretory nuclei; AH, anterior hypothalamic area; AP, area postrema; ArC, arcuate hypothalamic nucleus; BNST, bed nucleus of the stria terminalis; Bötzinger, Bötzinger complex; CeA, central amygdaloid nucleus; CVLM, caudal ventrolateral medulla; DMH, dorsomedial hypothalamic nucleus; DMNV, dorsal motor nucleus of the vagus nerve; DRN, dorsal raphe nucleus; GiA, gigantocellular reticular nucleus, alpha part; GiV, gigantocellular reticular nucleus, ventral part; HDB, nucleus of the horizontal limb of the diagonal band; IRt, intermediate reticular nucleus; isRT, isthmic reticular nucleus; LC, locus coeruleus; LH, lateral hypothalamic area; LM, lateral mammillary nucleus; LPB, lateral parabrachial nucleus; LPO, lateral preoptic area; LRt, lateral reticular nucleus; LSN, lateral septal nucleus; MPB, medial parabrachial nucleus; MPOA, medial preoptic area; mRt, medial raphe nucleus; MTu, medial tuberomammillary nucleus; MVPO, medioventral periolivary nucleus; NA, nucleus ambiguus; NTS, nucleus of the solitary tract; OVLT, organum vasculosum of the lamina terminalis; PAG, periaqueductal gray; PCRtA, parvicellular reticular nucleus, alpha part; PH, posterior hypothalamic area; PrBötzinger, pre‐Bötzinger complex; PSTh, parasubthalamic nucleus; PTg, pedunculopontine tegmental nucleus; RLi, rostral linear nucleus of the raphe; RMg, raphe magnus nucleus; RMM, rostromedial tegmental nucleus; RRF, retrorubral field; RTN, retrotrapezoid nucleus; RtTg, reticulotegmental nucleus of the pons; RVRG, rostral ventral respiratory group; RVLM, rostral ventrolateral medulla; SNR, substantia nigra, reticular part; SP5, spinal trigeminal nucleus; VDB, nucleus of the vertical limb of the diagonal band; VLPO, ventrolateral preoptic nucleus; VMH, ventromedial hypothalamic nucleus; VMPO, ventromedial preoptic nucleus; VP, ventral pallidum; VTA, ventral tegmental area.

To delineate the efferent targets of PVN^CRH^ neurons, we employed a Cre‐dependent anterograde viral tracing strategy by injecting AAV‐EF1α‐DIO‐EYFP into the PVN (Figure 5A). We observed that the continuous coronal sections of the virus transfection were confined to the PVN in 3 of 5 mice (Figure S3). Using the same viral strategy on wild‐type mice, no reporter gene expression was observed (Figure S4A,B). RNAscope‐FISH analyses confirmed that 71.5% of the 144 neurons counted were EYFP^+^ *Crh* ^+^ (Figure 5B), and immunohistochemical imaging demonstrated that EYFP^+^ somata were strictly confined within the boundaries of the PVN on the inject side (Figure 5C). Based on images of consecutive coronal sections, we observed that EYFP‐labeled axonal terminals were broadly distributed across multiple downstream brain regions (Figure 5D).

**FIGURE 6:**
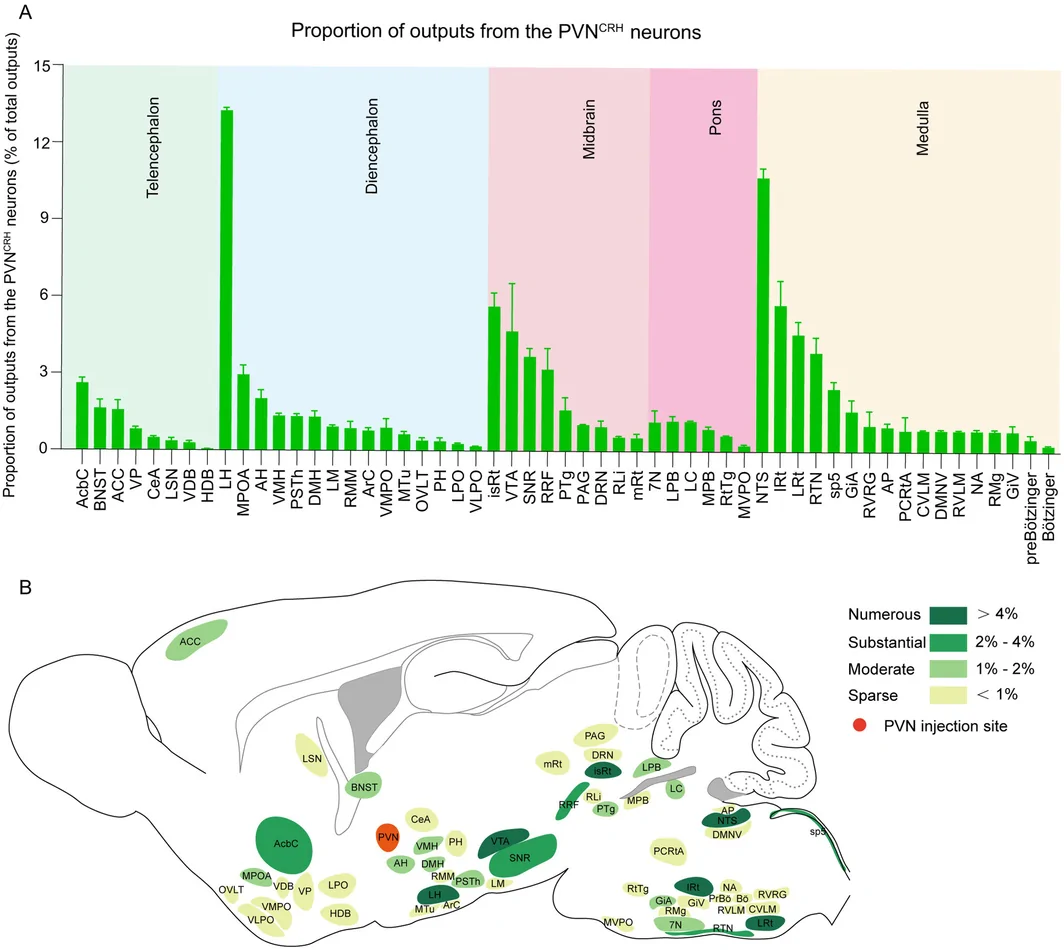
Quantitative analysis and functional classification of efferent projections from PVN^CRH^ neurons. (A) Quantitative analysis of the proportion of efferent projections from PVN^CRH^ neurons across the whole brain, expressed as a percentage of total efferent outputs. Brain regions are categorized into major divisions: Telencephalon, diencephalon, midbrain, pons, and medulla oblongata. Data are presented as mean ± SEM, *n* = 3 mice. (B) Sagittal schematic illustrating the whole‐brain distribution and density of efferent projections from PVN^CRH^ neurons. The color of each region represents its axonal projection density, with the PVN as the virus injection site (orange).

After excluding the PVN itself, we quantified axonal projection patterns in each target region by counting fluorescent puncta‐like enlargements associated with labeled fibers, while excluding clearly identifiable fibers of passage (Figure 5D). These signals were used to estimate the regional distribution of labeled axonal projections. Across the whole brain, axon terminals of PVN^CRH^ neurons were distributed in the telencephalon, diencephalon, midbrain, pons, and medulla oblongata. In the telencephalon, the nucleus accumbens core (AcbC) received the most prominent innervation (2.6% ± 0.2%). Within the diencephalon, the LH emerged as the dominant target, exhibiting the highest proportion of axons among all quantified nuclei (13.2% ± 0.1%). In the midbrain, the intermediate layer of the superior colliculus (isRt) accounted for the largest fraction of labeled terminals (5.6% ± 0.5%). In the pons, the facial nucleus (7N) was the major target (1.2% ± 0.5%), whereas the NTS received the densest innervation (10.7% ± 0.4%) in the medulla (Figure 6A). To provide an intuitive overview of output strength, all efferent nuclei were further categorized into four tiers based on their contribution to the total input population: numerous (> 4%), substantial (2%–4%), moderate (1%–2%), and sparse (< 1%) (Figure 6B).

Taken together, these data demonstrate that PVN^CRH^ neurons send widespread efferent projections not only to hypothalamic and brainstem nuclei governing innate and physiological states, but also to telencephalic and mesolimbic circuits involved in higher‐order cognitive and emotional processes. This projection architecture provides direct neuroanatomical evidence supporting a central role of PVN^CRH^ neurons in HPA axis–related stress responses and in the integration of emotional, autonomic, and behavioral states.

## 4 Discussion

This study provides a whole‐brain, monosynaptic input–output atlas of PVN^CRH^ neurons using cell type–specific rabies tracing combined with anterograde AAV labeling. We demonstrate that PVN^CRH^ neurons receive direct inputs from 55 nuclei, with a strong bias toward diencephalic and telencephalic sources, and send widespread projections to hypothalamic, limbic, and brainstem autonomic centers. These data define the anatomical framework by which PVN^CRH^ neurons couple stress‐ and emotion‐related signals to endocrine and autonomic effectors.

### 4.1 Comparison With Prior Connectomic Studies and Identification of Novel Circuitry

While the afferent and efferent projections of PVN^CRH^ neurons have been partially characterized using conventional tract‐tracing methods, our study provides several advances in terms of resolution, scope, and quantification. Previous work, largely in rats, established that PVN neurons receive inputs from a relatively circumscribed set of structures, including the brainstem (particularly the NTS and catecholaminergic cell groups), circumventricular organs, and local hypothalamic regions, while projecting heavily to the median eminence, brainstem autonomic nuclei, and spinal cord [34, 35, 36]. However, these foundational studies typically relied on non‐cell type‐specific tracers or lacked the Cre‐dependent genetic access to selectively target CRH‐expressing neurons.

By combining Cre‐dependent monosynaptic rabies tracing with anterograde AAV labeling in CRH‐Cre mice, we obtained a comprehensive and quantitative whole‐brain input–output map at the level of a single molecularly defined PVN subpopulation. This approach confirms many of the connections described in earlier work, but also reveals several undetermined or more specific inputs and outputs. For example, while the NTS has long been recognized as a major source of ascending catecholaminergic input to the PVN [37, 38], our quantitative analysis demonstrates that it is a substantial source of both ipsilateral and contralateral input to PVN^CRH^ neurons. In addition, our data highlight a prominent and previously underappreciated input from vCA1 of the hippocampus and from the AcbC. The hippocampus has been extensively studied for its role in HPA axis negative feedback, and the prevailing model emphasizes indirect influences via relays in the BNST and peri‐PVN region rather than direct monosynaptic projections onto PVN^CRH^ neurons [39, 40, 41]. Our finding of a vCA1 input suggests that, in addition to these indirect pathways, there may also be a more direct route by which hippocampal circuits modulate PVN^CRH^ activity, consistent with recent evidence for a functional hippocampus–PVN pathway that can attenuate acute stress responses [42]. Similarly, the identification of direct inputs from the AcbC—a region central to reward and motivation—provides an anatomical substrate for the direct influence of reward‐related signals on the HPA axis, which has been implicated in functional studies [22, 43].

Notably, our PVN^CRH^‐specific anterograde tracing is broadly consistent with earlier descriptions of PVN projections to brainstem autonomic nuclei such as NTS, RVLM, and related reticular regions [34, 35, 36], but further specifies their origin within the CRH population and reveals additional outputs to defined prefrontal, limbic, and respiratory‐related nuclei. Together, these comparisons indicate that our cell type‐specific connectome extends the current PVN projection map by identifying previously unresolved CRH‐specific pathways that will be functionally validated in the future.

### 4.2 PVN^CRH^ Neurons Linking Stress Appraisal to HPA Axis and Emotional Behavior

Our input–output data indicate that PVN^CRH^ neurons are embedded in a distributed forebrain–hypothalamic–brainstem network that contributes to control of stress and emotion. PVN^CRH^ neurons receive dense monosynaptic innervation from limbic and prefrontal regions, including the prelimbic cortex (PrL), LSN, BNST, central amygdaloid nucleus (CeA), medial amygdaloid nucleus (MeA), and AcbC, as well as from diencephalic nuclei such as PVT, LH, DMH, VMH, and vCA1. Many of these regions are key nodes in circuits that encode threat, anxiety, contextual memory, and reward [27, 44, 45, 46, 47, 48, 49]. The strong convergence from PrL, vCA1, and PVT suggests that PVN^CRH^ neurons can integrate cognitive appraisal and contextual information about stressors, while inputs from BNST, CeA, and MeA provide direct access to circuits processing fear and sustained anxiety. This organization is consistent with evidence that early‐life and chronic stress enhance PVN^CRH^ excitability and drive long‐lasting anxiety‐like behaviors, and that chemogenetic or pharmacological suppression of PVN^CRH^ activity exerts anxiolytic effects [27, 29, 50].

PVN^CRH^ neurons project back to several forebrain structures that provide input, including the BNST, CeA, MeA, and AcbC, as well as to the VTA and other mesolimbic regions. These reciprocal and feed‐forward connections offer a direct anatomical substrate by which PVN^CRH^ neurons can influence emotional valence, avoidance and approach behavior, and reward processing in parallel with their hormonal actions. Optogenetic activation of PVN^CRH^ neurons has been shown to induce conditioned place aversion and suppress reward‐seeking, whereas inhibition promotes place preference [43]. The efferent projections identified to prefrontal, amygdala, septal, and mesolimbic regions provide a structural correlate for these observations, indicating that PVN^CRH^ neurons can rapidly bias higher‐order circuits toward defensive and avoidance states during stress, while simultaneously engaging the HPA axis [19, 51].

Classically, PVN^CRH^ neurons have been viewed as the hypothalamic origin of the HPA axis, releasing CRH into the median eminence to drive pituitary ACTH and adrenal glucocorticoid secretion [27, 52]. Our connectivity map refines this view by showing how limbic and thalamic inputs associated with stress appraisal, controllability, and context converge directly onto PVN^CRH^ neurons. Taken together, these data support a model in which PVN^CRH^ neurons function as an integrative hub that links distributed stress‐appraisal circuits to coordinated endocrine and emotional responses.

### 4.3 PVN^CRH^ Neurons Coordinating Autonomic, Visceral, and Somatic Responses to Stress

Beyond their role in emotional regulation and HPA axis activation, our data suggest that PVN^CRH^ neurons are positioned to influence a broad range of autonomic and visceral functions. At the brainstem level, PVN^CRH^ projections densely innervate nuclei involved in cardiovascular and respiratory control, including the NTS, LPB, RVLM, CVLM [53, 54, 55, 56, 57], and respiratory pattern‐generating regions such as the pre‐Bötzinger and Bötzinger complexes [58, 59, 60]. These pathways provide an anatomical substrate for stress‐induced tachycardia, blood pressure elevation, and changes in breathing pattern that have been linked to PVN^CRH^ activity in functional studies [61, 62]. For example, activation of PVN^CRH^ neurons increases arterial pressure and heart rate, and their hyperactivity in hypertensive models is associated with enhanced sympathetic outflow, whereas optogenetic or chemogenetic inhibition of these neurons lowers blood pressure and renal sympathetic nerve activity [23, 63, 64]. The strong descending inputs to the NTS and ventrolateral medullary nuclei offer a direct structural explanation for such effects.

PVN^CRH^ neurons also project to hypothalamic and brainstem regions implicated in metabolic and gastrointestinal regulation, including the VMH, LH, DMNV, and other autonomic premotor areas. These connections are compatible with findings that PVN^CRH^ neurons rapidly initiate stress‐induced hyperglycemia via hypothalamic mechanisms and contribute to stress‐related gastric dysmotility through projections to dorsal vagal motor pathways [25, 65]. Furthermore, descending projections to autonomic and catecholaminergic nuclei provide a possible route by which PVN^CRH^ neurons influence immune and inflammatory processes, as suggested by reports that their activation can modulate peripheral inflammation via sympathetic pathways [26]. In this context, the PVN^CRH^ network can be seen as coordinating the autonomic and visceral components of the stress response in parallel with endocrine HPA activation, rather than acting solely as an upstream trigger of glucocorticoid release.

Our quantitative analysis of inputs also revealed a modest but consistent ipsilateral dominance, with approximately 60% of input neurons located on the injected side and 40% contralaterally. Although we did not formally quantify lateralization of efferent projections, labeled axons were observed bilaterally in many downstream autonomic and visceral centers. This biased yet bilaterally organized architecture suggests that PVN^CRH^ neurons can coordinate symmetric autonomic outputs while integrating slightly asymmetric forebrain and hypothalamic inputs. Whether such lateralization contributes to differences in stress coping strategies or in the control of cardiovascular and visceral responses remains to be tested. Together, these output patterns indicate that PVN^CRH^ neurons occupy a central position linking emotional and endocrine stress responses to coordinated changes in cardiovascular, respiratory, metabolic, and gastrointestinal function. Our connectivity map therefore provides a structural basis for understanding how dysregulated PVN^CRH^ activity could contribute not only to stress‐related psychiatric symptoms, but also to stress‐associated autonomic and somatic comorbidities.

### 4.4 Limitations

Several methodological limitations of this study warrant consideration. First, our RNAscope analysis showed that approximately 82%–88% of reporter‐positive neurons in the PVN co‐expressed *Crh* mRNA, indicating that a small fraction (12%–18%) of labeled cells may not be CRH neurons. This is consistent with previous characterizations of the CRH‐IRES‐Cre line and may reflect transient developmental CRH expression leading to permanent recombination, or technical detection thresholds [66, 67, 68]. Thus, our connectome represents predominantly, but not exclusively, PVN^CRH^ neurons. Second, this study was conducted exclusively in male mice to minimize estrous cycle variability; however, stress responses and HPA regulation are sexually dimorphic, and our findings should be considered a male‐specific atlas requiring future validation in females [34, 69]. Third, the irreversible nature of Cre/loxP recombination labels neurons with a history of *Crh* expression, which may contribute to discrepancies with studies reporting minimal direct hippocampal input to adult PVN^CRH^ neurons [42, 70]. Fourth, anatomical tracing and quantification of input neuron numbers cannot be directly equated with functional influence. Our analyses describe the relative distribution of labeled cells and axonal puncta‐like signals, but do not account for synaptic density, synaptic strength, or the location of synapses on the postsynaptic neuron. In particular, the rostro‐caudal distribution analysis was normalized to the total number of labeled input neurons in each animal, rather than to the total number of neurons or cells present within each coronal section. Thus, this analysis should be interpreted as a descriptive summary of where labeled input neurons were detected along the rostro‐caudal axis, and not as a measure of preferential input density within individual coronal planes. Regions with sparse anatomical input may still exert strong physiological effects, whereas regions with numerous labeled neurons may have heterogeneous or predominantly modulatory actions. The functional relevance of the connections identified here will therefore require validation using circuit‐specific manipulations and in vivo recordings. In addition, our identification of axonal puncta‐like enlargements in the anterograde dataset is based on light‐microscopic morphology and should be regarded as anatomical features of labeled fibers rather than functionally confirmed synaptic boutons or synaptic sites.

## 5 Conclusion

By combining monosynaptic rabies tracing with Cre‐dependent anterograde labeling, this study defines the whole‐brain input–output architecture of PVN^CRH^ neurons at cellular resolution. Beyond documenting widespread connectivity, our data show that PVN^CRH^ neurons are positioned at the intersection of limbic stress‐appraisal circuits, hypothalamic internal‐state centers, and brainstem autonomic and respiratory nuclei. This organization supports a view of PVN^CRH^ neurons not only as the hypothalamic origin of the HPA axis but as a central node that links psychological and contextual information to coordinated endocrine, emotional, and autonomic responses. The connectivity framework provided here offers a structural basis for future circuit‐level studies of how dysregulated PVN^CRH^ activity contributes to stress‐related psychiatric and cardiometabolic disorders.

## Author Contributions

Study design and manuscript drafting/revising: S.W. and X.W. Running of the experiments, acquisition of the data, and figure drawing: Z.Y., D.Z., X.C., Y.W., Y.L. Acquisition of the data and statistical analysis: C.G., Y.Y., H.Z., Y.S. All authors have read and approved the final version of the manuscript.

## Funding

This work was supported by the Natural Science Foundation of China (U23A20431) and Natural Science Foundation of Hebei Province for Innovative Research Group Project (H2025206897) to S.W.; the China Postdoctoral Science Foundation (2025M782604) and Postdoctoral Science Foundation of Hebei Medical University (Pharmacology Postdoctoral Station) to X.W.; Hebei Medical University Natural Science Foundation for Outstanding PhD Student to Z.Y. (YBKJ202502).

## Ethics Statement

All experiments were performed in accordance with the Guide for the Care and Use of Laboratory Animals and were approved by the Animal Care and Ethical Committee of Hebei Medical University (Hebmu‐P2023052).

## Conflicts of Interest

The authors declare no conflicts of interest.

## Supporting information


**Figure S1:** Validation of Cre‐dependent specificity of the retrograde tracing system.
**Figure S2:** PVN injection sites across individual mice used for retrograde tracing.
**Figure S3:** PVN injection sites across individual mice used for anterograde tracing.
**Figure S4:** Validation of Cre‐dependent specificity of the anterograde tracing system.

## Data Availability

The data that support the findings of this study are available from the corresponding author upon reasonable request.
