# Supplementary material for: Whole‐Brain Monosynaptic Inputs and Outputs of Corticotropin‐Releasing Hormone Neurons of the Paraventricular Nucleus of the Hypothalamus in Male Mice
Source: CNS Neurosci Ther. 2026 Aug 30;32(9):e71127. doi: 10.1002/cns.71127 (PMC13527161; doi:10.1002/cns.71127)
Supplement: Supplementary file 1 [file CNS-32-e71127-s001.docx]

**Whole-brain monosynaptic inputs and outputs of corticotropin-releasing hormone neurons of the paraventricular nucleus of hypothalamus in male mice**

Ziteng Yue^1, *^, Danyang Zhao^1, *^, Xinxin Chen^1^, Yousheng Zhang^1^, Heng Zhang^1^, Yinchang Wang^1^, Yuhang Liu^1^, Yuxin Yang^1^, Can Geng^1^, Yimeng Song^1^, Xiaoyi Wang^1, 2, #^, Sheng Wang^1, 2, 3, #^

**Supplementary Files include: Supplementary Figures 1-4**

**Supplementary Figure 1**

**Supplementary Figure 1. Validation of Cre-dependent specificity of the retrograde tracing system.** (A) Schematic illustration of the viral injection strategy in wild-type (C57BL/6J) mice. Helper viruses (AAV-EF1α-DIO-TVA-GFP and AAV-EF1α-DIO-RG) were co-injected into the PVN, followed 14 days later by injection of the pseudotyped rabies virus (RV-EnvA-ΔG-tdTomato) at the same coordinates. All viral injections were performed using the same volumes and coordinates as in CRH-Cre mice. (B) Representative coronal section through the PVN showing DAPI nuclear staining (blue) and the absence of both helper virus-derived GFP (green) and rabies virus-derived tdTomato (red) expression. Scale bar, 100 μm.

**Supplementary Figure 2**

**Supplementary Figure 2. PVN injection sites across individual mice used for retrograde tracing.**  Representative serial coronal sections through the PVN from five CRH-Cre mice (animal IDs: A51, A53, A54, A55, A58) that received sequential injections of helper viruses (AAV-DIO-TVA-GFP and AAV-DIO-RG) and RV-EnvA-ΔG-tdTomato into the PVN, with accurate viral targeting. Starter neurons (GFP⁺tdTomato⁺, appearing yellow in merged images) were strictly confined within the PVN boundaries in all five mice. A total of eight animals (A51–A58) received injections; three mice were excluded from analysis due to inaccurate injection placement. Scale bars, 100 μm.

**Supplementary Figure 3**

**Supplementary Figure 3. PVN injection sites across individual mice used for anterograde tracing.**  Representative serial coronal sections through the PVN from three CRH-Cre mice (animal IDs: A83, A84, A86) that received the Cre-dependent anterograde tracer rAAV9-EF1α-DIO-EYFP and exhibited accurate viral targeting. EYFP⁺ neurons (green) were strictly confined within the PVN boundaries in all three mice. A total of five animals (A83–A87) received injections; two mice were excluded from analysis due to inaccurate injection placement. Scale bar, 100 μm

**Supplementary Figure 4**

**Supplementary Figure 4. Validation of Cre-dependent specificity of the anterograde tracing system.** (A) Schematic illustration of the viral injection strategy in wild-type (C57BL/6J) mice. The Cre-dependent anterograde tracer rAAV9-EF1α-DIO-EYFP was injected into the PVN using the same coordinates and volume as in CRH-Cre mice. (B) Representative coronal section through the PVN showing DAPI nuclear staining (blue) and absence of EYFP expression (green). No EYFP⁺ cell bodies were detected within the PVN. Scale bar, 100 μm.
